# Acute Cauda Equina Syndrome Caused by Epidural Steroid Injection in the Setting of a Spinal Dural Arteriovenous Fistula

**DOI:** 10.7759/cureus.21752

**Published:** 2022-01-30

**Authors:** Kaitlyn L Slimp, Lara N Martinez, Jeffrey A Nielson, Roy L Johnson

**Affiliations:** 1 Emergency Medicine, Kettering Health Network, Dayton, USA

**Keywords:** av epidural fistual, atrio-esophageal fistula, emergency medicine, epidural injection, cauda equina syndrome

## Abstract

We present a case of acute cauda equina syndrome caused by an epidural steroid injection in the setting of a previously undiagnosed spinal dural arteriovenous fistula (SDAVF). Our patient was a 61-year-old man who presented to the emergency department with low back pain, inability to walk, paresthesias of his bilateral lower extremities, bowel and bladder incontinence, and saddle anesthesia. Physical examination revealed weakness and decreased sensation of the lower extremities as well as poor rectal tone and urinary retention. Magnetic resonance imaging (MRI) revealed evidence of spinal cord edema in the T9-10 region and a probable SDAVF with secondary distal thoracic cord ischemia. This case highlights the importance of prompt recognition of cauda equina syndrome in the emergency department, expedient imaging, and efficient transfers of care, which allowed this patient to quickly undergo necessary surgery that led to an almost complete recovery. It also highlights the importance of recognizing subtle changes on lumbar MRI.

## Introduction

Spinal dural arteriovenous fistula (SDAVF) is a rare condition in which an abnormal connection exists between the arteries and veins surrounding the spinal cord [1]. This is often initially asymptomatic but can begin to cause symptoms such as lower extremity weakness or sensory deficits, back or leg pain, and bowel or bladder dysfunction. However, symptoms are usually vague, making early diagnosis difficult.

## Case presentation

**Figure 1:**
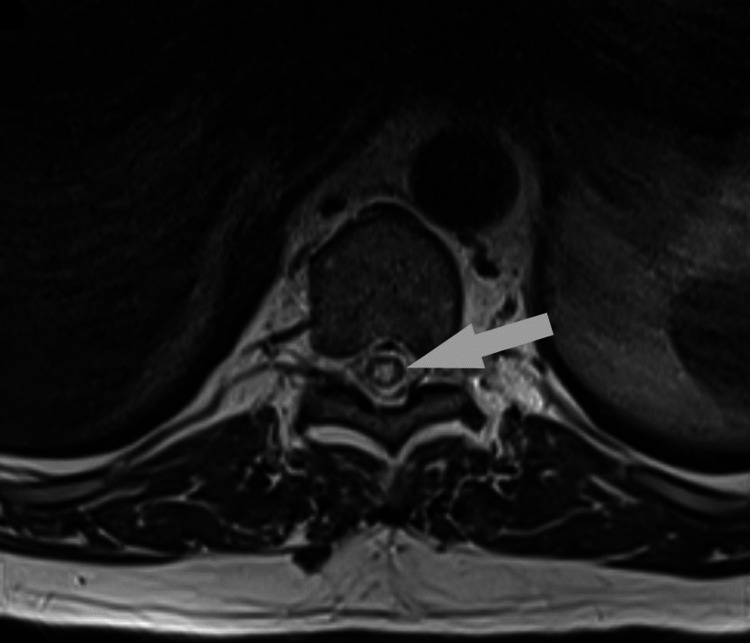
Axial T2 image showing cord edema

**Figure 2:**
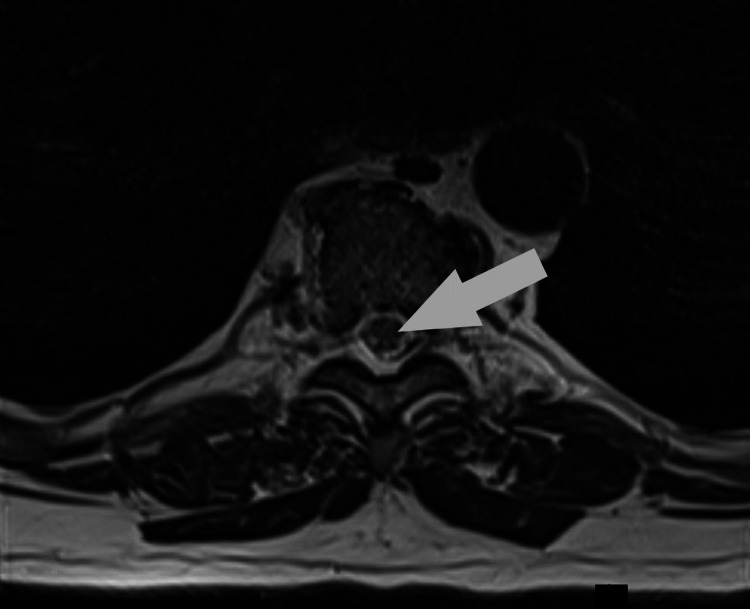
Axial T2 image showing central cord ischemia

**Figure 3:**
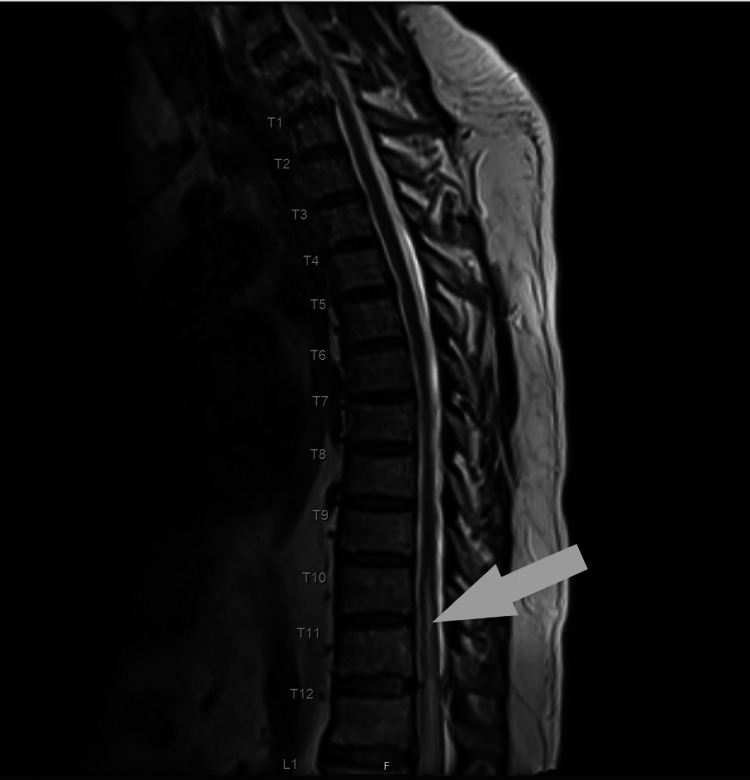
Sagittal T2 image showing central cord ischemia

We present a case of a 61-year-old Caucasian patient with spinal degenerative disease and an unrecognized SDAFV who had sudden onset of cauda equina syndrome following an interlaminar, fluoroscopy-guided epidural steroid injection. He presented with low back pain, inability to walk, paresthesias of his bilateral lower extremities, bowel and bladder incontinence, and saddle anesthesia. He denied any history of intravenous drug abuse, fever, chills, or other infectious symptoms. He denied prior surgery. His vital signs were all unremarkable. His physical examination revealed decreased sensation and 4/5 strength in his bilateral lower extremities, as well as severely diminished rectal tone. Bladder scan revealed urinary retention with greater than 500 cc of urine drained after Foley catheterization. Lab evaluation was unremarkable except for a urinalysis showing a few white blood cells and bacteria with no nitrites. A urine culture later grew *Streptococcus viridans*. Magnetic resonance imaging (MRI) revealed evidence of spinal cord edema in the T9-10 region (see Figures 1-3) and a probable SDAVF with secondary distal thoracic cord ischemia. Comparison to prior demonstrated that there was subtle prior abnormality-nonspecific and potentially within the range of normal.

**Figure 4:**
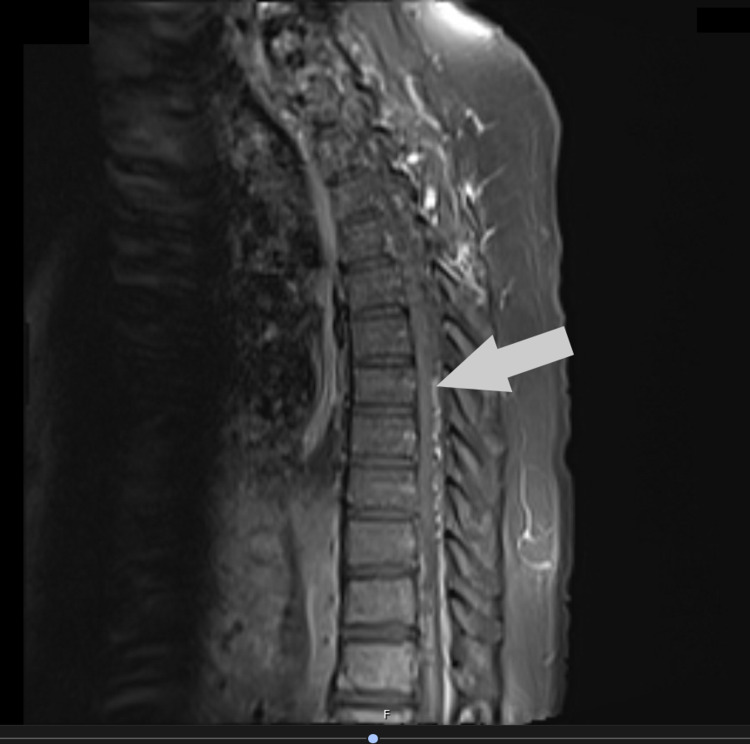
Sagittal T2 image with contrast showing arteriovenous malformation

Spinal cord angiography confirmed the arteriovenous malformation as seen in Figure 4. 

The patient was taken for laminectomy with resection of the SDAVF. The patient’s neurologic symptoms improved almost immediately and he was discharged home nine days later. The case was complicated only by urinary retention, which resolved at eight months.

## Discussion

SDAVF is a rarely diagnosed phenomenon that is usually found in elderly men. Though rare, they are the most frequent vascular malformation of the spine [1]. Most patients present with sensory loss in the lower extremities, weakness, and radiculopathy. Most patients experience gradual onset of these symptoms over months.

Small SDAFVs are not always clearly seen on MRI without angiography. This SDAFV went unrecognized initially, and only after the patient developed post-procedural symptoms more sensitive imaging was performed. This highlights the need for careful appreciation of nonspecific findings on imaging.

Our patient was unique in that he had sudden onset of symptoms exacerbated by an epidural steroid injection. We found two cases in the literature with similar symptoms [2,3], but both experienced a gradual onset of symptoms. There are several different treatment modalities to definitively treat SDAFV. These include surgical resection and arterial embolization. In general, embolization tends to have a higher recurrence rate, making surgical resection a more favorable definitive treatment [4]. Our patient was very fortunate to undergo surgical resection on the day of presentation. He had immediate improvement in his symptoms and was able to be discharged the next week with only some residual urinary retention, which also later resolved.

## Conclusions

This case reveals a classic presentation of cauda equina syndrome brought on by a very uncommon cause-exacerbation of a previously undiagnosed SDAVF worsened by routine spinal injection. Prompt recognition of cauda equina syndrome, expedient imaging, and efficient transfers of care allowed this patient to quickly undergo necessary surgery that led to an almost complete recovery. At six months, the patient’s only residual symptoms were related to a neurogenic bladder requiring self-catheterization. This had resolved entirely by eight months post-operation.
